# Different Biochemical Compositions of Particulate Organic Matter Driven by Major Phytoplankton Communities in the Northwestern Ross Sea

**DOI:** 10.3389/fmicb.2021.623600

**Published:** 2021-01-21

**Authors:** Naeun Jo, Hyoung Sul La, Jeong-Hoon Kim, Kwanwoo Kim, Bo Kyung Kim, Myung Joon Kim, Wuju Son, Sang Heon Lee

**Affiliations:** ^1^Department of Oceanography, Pusan National University, Busan, South Korea; ^2^Division of Ocean Sciences, Korea Polar Research Institute, Incheon, South Korea; ^3^Division of Life Sciences, Korea Polar Research Institute, Incheon, South Korea; ^4^Department of Polar Science, University of Science and Technology, Daejeon, South Korea

**Keywords:** phytoplankton, biomolecular composition, amino acid composition, food quality, Ross Sea

## Abstract

Marine particulate organic matter (POM) largely derived from phytoplankton is a primary food source for upper trophic consumers. Their biochemical compositions are important for heterotrophs. Especially, essential amino acids (EAAs) in phytoplankton are well known to have impacts on the survival and egg productions of herbivorous zooplankton. To estimate the nutritional quality of POM, the biochemical compositions [biomolecular and amino acid (AA) compositions] of POM were investigated in the northwestern Ross Sea during the late austral summer in 2018. Carbohydrates (CHO) accounted for the highest portion among different biomolecules [CHO, proteins (PRT), and lipids (LIP)] of POM. However, the higher contribution of PRT and lower contribution of CHO were observed in the southern section of our study area compared to those in the northern section. The spatial distribution of total hydrolyzable AAs in POM was considerably influenced by phytoplankton biomass, which indicates that the main source of particulate AA was generated by phytoplankton. Our results showed that the relative contribution of EAA to the total AAs was strongly associated with EAA index (EAAI) for determining protein quality. This result indicates that higher EAA contribution in POM suggests a better protein quality in consistency with high EAAI values. In this study, variations in the biochemical compositions in POM were principally determined by two different bloom-forming taxa (diatoms and *Phaeocystis antarctica*). The southern region dominated majorly by diatoms was positively correlated with PRT, EAA, and EAAI indicating a good protein quality, while *P. antarctica-*abundant northern region with higher CHO contribution was negatively correlated with good protein quality factors. Climate-driven environmental changes could alter not only the phytoplankton community but also the physiological conditions of phytoplankton. Our findings could provide a better understanding for future climate-induced changes in the biochemical compositions of phytoplankton and consequently their potential impacts on higher trophic levels.

## Introduction

Marine particulate organic matter (POM) is derived from a variety of living and non-living sources, including detritus matter, bacterial cells, and phytoplankton (Volkman and Tanoue, 2002). Although the relative importance of these diverse sources cannot be clarified, phytoplankton is definitely the most important part of marine POM in surface waters (Riley, 1971; Kharbush et al., 2020). POM largely derived from phytoplankton plays a significant role in linking the primary producers to herbivores as a crucial food source (Dzierzbicka-Głowacka et al., 2010; Lowe et al., 2014; Andersson et al., 2017) and potential carbon export to the deep ocean (Ducklow et al., 2001; Basu and Mackey, 2018). Biochemical properties of POM, especially biomolecular and amino acid (AA) compositions, are useful indicators of nutritional quality for higher trophic consumers (Dell’Anno et al., 2000; Lee et al., 2004; Bhavya et al., 2019). The various biomolecular components, including carbohydrates (CHO), proteins (PRT), and lipids (LIP), are generated through photosynthetic assimilation of dissolved inorganic carbon into organic compounds within phytoplankton (Fernández-Reiriz et al., 1989; Fichez, 1991). The relative contribution of the biomolecular compounds produced by phytoplankton is tightly linked to the prevailing environmental conditions (e.g., availability of nutrients and light), major phytoplankton groups, and the growth phase of phytoplankton (Ahn et al., 2019; Bhavya et al., 2019 and the references therein). Thus, the biomolecular composition of phytoplankton has also been considered a suitable indicator of the physiological responses of phytoplankton to the limitation of macro and micronutrients (i.e., bioavailable N, P, Si, and Fe) (Morris et al., 1974; Sterner and Elser, 2002; Saito et al., 2008; Moore et al., 2013) and light stress (Morris et al., 1974; Smith and Morris, 1980; Sunda and Huntaman, 1997; Klausmeier et al., 2008) which is consequently connected with their nutritional quality for higher trophic consumers (Bhavya et al., 2019).

On the one hand, it is well known that AAs are the building blocks of different biomolecules which are mainly peptides and PRT (Kolmakova and Kolmakov, 2019; Shields et al., 2019). Previous studies have shown that compositional changes in AAs are related to the degradation state of POM, phytoplankton community structure, and growth phase of phytoplankton (Hecky et al., 1973; Kolmakova and Kolmakov, 2019; Shields et al., 2019). Therefore, these compositional changes of AAs have widely been used to indicate the organic matter degradation (Cowie and Hedges, 1992; Mente et al., 2002; Becker and Richmond, 2004) and protein quality (Oser, 1959; Mente et al., 2002; Becker and Richmond, 2004). The degradation index (DI) based on the changes in the relative abundance of each AA to total AAs during organic matter diagenesis can be applied to estimate the degradation degree of POM in sediment as well as sinking particles (Dauwe and Middelburg, 1998; Dauwe et al., 1999; Le Moigne et al., 2017). Among different AAs, essential AAs (EAAs) cannot be synthesized *de novo* by most heterotrophic organisms and must therefore be fulfilled by prey to meet consumer’s nutritional needs for their growth and reproduction (Muller-Navarra, 1995; Kleppel et al., 1998; Kolmakova and Kolmakov, 2019). However, nutritional quality for higher trophic levels comprises not only the quantity of EAA but also balance in individual EAAs (Müller-Navarra, 2008). Hence, the EAA index (EAAI) allows us to evaluate the protein quality in terms of the AA composition of POM as consumers’ diets.

The Ross Sea is one of the most productive regions in the Southern Ocean and thus supporting considerable standing stocks of apex predators such as penguins, seals, and whales (Nelson et al., 1996; Pinkerton et al., 2010). In 2016, this region was established as a massive Marine Protected Area (MPA) safeguarding 1.55 million km^2^ of ocean bordering Antarctica from ice edge to deep ocean by the Commission for the Conservation of Antarctic Marine Living Resources (CCAMLR). Of that, cape Hallett located at the northern Victoria Land is one of the specially protected areas and large populations of breeding penguins relevant to large aggregations of krill as their primary food source (Lyver et al., 2011). The marine top predators (e.g., penguins, seals, and whales) depend directly or indirectly on organic matter by photosynthetic microalgae since the quantity and quality of POM produced by phytoplankton have consequences for the entire marine ecosystem of the Ross Sea through bottom-up processes (Oksanen and Oksanen, 2000; Gruner et al., 2008). Indeed, the Ross Sea food webs are supported at their foundation by phytoplankton comprising of two key algal groups: diatoms and haptophytes, particularly *Phaeocystis antatarctica* (DiTullio and Smith, 1996; Alderkamp et al., 2012; Smith et al., 2014; Mangoni et al., 2017). The relative abundance of major two phytoplankton communities varies with spatial and temporal patterns in the Ross Sea and subsequently can have significant influences on the spatial and temporal diet variability of higher trophic levels (trophodynamics) (Young et al., 2015b; Mangoni et al., 2019). According to current climate trends, the Ross Sea is expected to experience extreme warming, decreased sea ice concentrations, and shallower mixed layers throughout the next century (Bracegirdle et al., 2008; Ainley et al., 2010; Bracegirdle and Stephenson, 2012). The changes in the predominant phytoplankton community and physiological status of phytoplankton caused by this predicted climate change (e.g., increase in sea surface temperatures, decreases in the mixed layer depths, sea ice concentrations, and macronutrient concentrations; Rickard and Behrens, 2016) can have profound implications on diet variability of higher trophic levels (Smith et al., 2003; Smetacek et al., 2004; Tang et al., 2008). Therefore, the aims of the paper were to (1) investigate biochemical compositions (biomolecular and AA compositions) of POM derived mainly phytoplankton and main factors in controlling the relative dominance of these biochemical compositions and (2) evaluate physiological conditions of phytoplankton and potential food quality as prey for consumers.

## Materials and Methods

### Study Sites and Sampling

**FIGURE 1:**
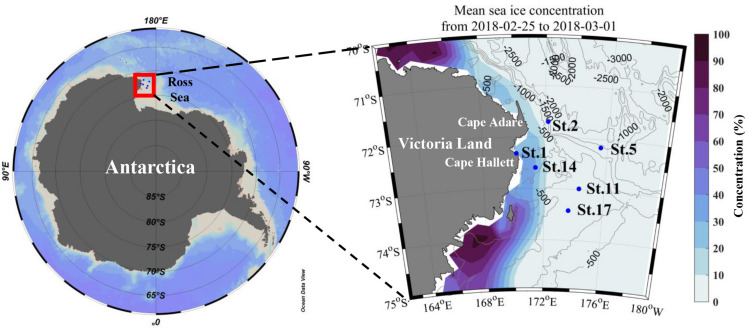
Map of the study area and sampling stations (with depth contours in meters). Mean sea ice concentration (%) data during the sampling period from Nimbus-7 SMMR and DMSP SSM/I-SSMIS Passive Microwave data provided by National Snow and Ice Data Center.

The field survey was performed closely to Cape Adare and Cape Hallett during the Ross Sea Marine Projected Area Expedition (ANA08C; from 25 February to 1 March 2018) in Antarctica on the IBR/V Araon (Figure 1). The vertical temperature and salinity profiles were collected using a conductivity-temperature-depth (CTD) recorder (SeaBird Electronics Inc., SBE 911 plus). At all sampling stations, discrete water samples for biological and chemical analyses were obtained from three different light levels (100, 30, and 1% light penetration depths which were estimated from the Secchi depth) employing CTD/rosette sampler attached to 24-10 L Niskin bottles. The depth of the euphotic zone (Z_eu_) was defined as the depth at which 99% of the surface irradiance is attenuated (Kirk, 1985) and estimated using a Secchi disk. The mixed layer depth (Z_m_) was defined as the depth where a change of 0.01 kg m^–3^ in potential density (σ_t_) from the stable surface layer value (Smith et al., 2000; Asper and Smith, 2019).

### Particulate Organic Carbon (POC), Nitrogen (PON), and Stable Carbon Isotopes (δ^13^C) Analyses of POM

For the analyses of POC, PON, and δ^13^C, 0.3 L of sampled sample was filtered through Whatman GF/F filter (25 mm, 0.7 μm pore) and immediately stored at −80°C. The filtered samples were acidified over fuming HCl to eliminate inorganic carbon before further analysis. Then, the filters were analyzed for carbon and nitrogen contents and isotope ratios using a Finnigan Delta + XL mass spectrometer at the stable isotope laboratory of the University of Alaska Fairbanks, United States.

### Major Inorganic Nutrients, Chlorophyll a, and Other Phytoplankton Pigments Analyses

Samples for the determination of dissolved inorganic nutrients (phosphate, nitrate + nitrite, ammonium, and silicate) were collected directly from the Niskin rosette into 50 mL conical tubes and immediately stored at 4°C until analysis within 24 h. Nutrient concentrations were measured on board with a QuAAtro Continuous Segmented Flow Analyzer (Seal Analytical, Norderstedt, Germany) using standard colorimetric methods according to the “QuAAtro Applications.”

Water samples (0.3 L) were filtered through 25 mm GF/F filter papers (Whatman, 0.7 μm pore) to measure the total chlorophyll a (chl-*a*) concentration. Sequential filtrations were performed to determine each size-fractionated chl-*a* concentration (>20, 5–20, and <5 μm). First, 0.5 L of seawater was filtered through the Polycarbonate Track Etched (PCTE) membrane 20 μm filter (GVS, 47 mm). Then, the filtrate was passed through the PCTE membrane 5 μm filter (Whatman, 47 mm) and 47 mm GF/F filters (Whatman, 0.7 μm pore) in sequence. The chl-*a* pigment was extracted by submerging filtered samples in 90% acetone for 24 h in dark and cold conditions (Parsons et al., 1984). The chl-*a* fluorescence was measured onboard using the pre-calibrated Trilogy fluorometer (Turner Designs, United States).

Phytoplankton pigment analysis using a high-performance liquid chromatography (HPLC) system can be used to quantify concentrations of each pigment that were determined by measuring the integrated peak area based on the method of Zapata et al. (2000). The pigments on the filtered samples (1 or 2 L of seawater) were extracted in 5 mL of 100% acetone with canthaxanthin (internal standard) for 24 h in dark at 4°C. The extract was filtered through a 0.2 μm Advantec syringe filter. HPLC measurements were performed on an Agilent 1200 HPLC system (Agilent infinite 1260, Agilent, United States) and the separating column was used Zobrax Eclipse XDB C8 column (250 × 4.6 mm, 5 μm, Agilent Technologies). The same analysis procedures of Kang et al. (2018) were performed for quantifying of pigment concentrations. As suggested by Mackey et al. (1996), the contribution of various phytoplankton classes could be estimated by the ratio of each diagnostic pigment to total chl-*a* using the CHEMTAX program. The initial pigment ratio to chl-*a* for each mark pigment used in the CHEMTAX program was modified by Mackey et al. (1996); Wright et al. (1996), and DiTullio et al. (2011).

### Biomolecular Composition of Phytoplankton

Water samples were obtained from three light depths (100, 30, and 1%) for biomolecular compositions (CHO, PRT, and LIP) of phytoplankton and filtered through a 47 mm GF/F filter. The filters were stored at −80°C for spectrometric analysis using a UV–visible spectrometer (Hitachi UH-5300, Japan) to measure each biomolecular concentration. CHO concentration was determined using the phenol-sulfuric method according to Dubois et al. (1956). After 1 mL of deionized water was added to the polypropylene tube containing the filtered sample, samples were ultrasonicated for 20 min for CHO extraction. 1 mL of 5% phenol reagent was additionally added, and the extracted samples were kept at room temperature for 40 min. The CHO after reaction with concentrated sulfuric acid were quantified by measuring the absorbance at 490 mm and then calculated from the calibration glucose standard (1 mg mL^–1^, SIGMA) curve. To measure PRT concentration based on Lowry et al. (1951), 1 mL of deionized water and 5 mL of alkaline copper solution (a mixture of 2% Na_2_CO_3_ in 0.1 N NaOH with 0.5% CuSO_4_⋅5H_2_O in 1% sodium or potassium tartrate; 50:1, v:v) were added into vials with the filtered samples for PRT extraction. After 20 min of ultrasonication, 0.5 mL of diluted Folin-Ciocalteu phenol reagent (1:1, v:v) was added into sample vials for the colorimetric reaction. The absolute concentration of PRT was calculated from the absorbance at 750 nm comparing with a protein standard solution (2 mg mL^–1^, SIGMA). The total LIP was extracted by chloroform and methanol (1:2, v:v) according to the modified method of Bligh and Dyer (1959) and Marsh and Weinstein (1966). The absorbance at 360 nm was expressed as tripalmitin equivalents. More detailed methods are explained in Bhavya et al. (2019).

### Amino Acid Composition Analysis

Samples for the analysis of total particulate hydrolyzable AAs (PAAs) were collected from three light depths (100, 30, and 1%) at six stations. Water samples (1 L) from each station were passed through 47 mm GF/F filters (Whatman, 0.7 μm pore) and then frozen at −80°C for later analysis. Acid hydrolysis was performed using the modified methods of Lobbes et al. (1999) and Bartolomeo and Maisano (2006). A filtered paper is transferred into the 5 mL reaction vial containing 2 mL HCl (6 M) and 10 μL ascorbic acid (11 mM). The vials were capped tightly after flushing with N_2_ gas and then moved into a pre-heated heating block at 110°C for 24 h. After acidic hydrolysis, hydrolysates were cooled at room temperature and filtered through 0.2 μm PTFE syringe filters (Advantec, Tokyo, Japan). Each remaining liquid was evaporated to dryness using a nitrogen evaporator at 60°C. The dried residues were reconstituted with 200 μL of 0.1 N HCl and transferred into glass vials for analysis. Samples were analyzed using HPLC (Agilent 1260 Infinity, Germany) equipped with an autosampler, a Zorbax-Eclipse AAA column (4.6 × 250 mm, 5 μm), and UV/VIS detector (338 and 262 nm). In the pre-column method, the samples and AA standard solutions were automatically derivatized with ortho-phthalaldehyde (OPA) and 9-fluorenylmethyl chloroformate (FMOC) by programming autosampler according to Agilent Application note (Henderson et al., 2000). The column temperature was maintained at 40°C with a flow rate of 1.5 mL/min. The mobile phase A contained 40 mM sodium phosphate (di-basic) with 0.1% phosphoric acid and mobile phase B was acetonitrile/methanol/deionized water (45:45:10, v:v:v). AA standard mixture with 21 L-AAs and L-norvaline (surrogate standard) was prepared for AA identification and quantification. AA standard solutions contained 22 L-AAs: Aspartic acid (ASP), Glutamic acid (GLU), Asparagine (ASN), Serine (SER), Glutamine (GLN), Histidine (HIS), Glycine (GLY), Threonine (THR), Arginine (ARG), Alanine (ALA), Tyrosine (TYR), Cystine (CY2), Valine (VAL), Methionine (MET), Tryptophan (TRP), Phenylalanine (PHE), Isoleucine (ILE), Leucine (LEU), Lysine (LYS), Hydroxyproline (HYP), Proline (PRO) and Norvaline (NVA). Hydroxyproline (HYP), and proline (PRO) could not be quantified because of their low responses and high detection limits in our HPLC. A representative chromatogram for the mixed standard is shown in Supplementary Figure S1. Each linear relationship for the four-point calibration curve of individual AAs was obtained with a correlation coefficient being above 0.999. The relative standard deviations of peak areas for each AA in each point ranged from 1.6 to 9.5% (*n* = 3) for measurement precisions. Before injecting, 20 μL of norvaline as a surrogate standard has added a sample of each vial and each sample was injected twice for HPLC analysis. Peak areas of AA measured that the average value of three blanks was subtracted from each sample analyzed. Then, individual AA concentrations in injected samples were calculated using the slope of the calibration curve of each AA and the known concentration of Norvaline. Glutamine (GLN) and asparagine (ASN) were quantified as glutamic acid (GLU) and aspartic acid (ASP) because glutamine (GLN) and asparagine (ASN) react into glutamic acid (GLU) and aspartic acid (ASP) during hydrolysis, respectively. Moreover, tryptophan (TRP) and cystine (CY2) are omitted from our AA results since they are fully or partially destroyed during acid hydrolysis. Therefore, the concentration of each remaining AA was expressed as a mole percentage (mol%) of the total AA.

### Amino Acid Index Calculations

The quantitative DI for POM was calculated using mol% AA composition and the factor coefficient of Dauwe et al. (1999). According to Dauwe et al. (1999), this index could reflect the reactivity of POM as degradation proceeds. DI was estimated using this equation derived by Dauwe et al. (1999).

$$DI=\sum_{i}\left[\frac{var_{i}-AVGvar_{i}}{STDvar_{i}}\right]\times fac\cdot coef_{i}$$

In this equation, var_*i*_ is the mol% of the individual AA, *AVGvar*_*i*_ and *STDvar*_*i*_ are the mean and standard deviation of the AA mol% in a given dataset, and *fac⋅coef_*i*_* is the factor coefficient in Dauwe et al. (1999).

Individual AA of each sample is divided into EAA and non-EAA (NEAA): nine essential (histidine, threonine, arginine, valine, methionine, phenylalanine, isoleucine, leucine, and lysine) and six non-essential (aspartic acid, glutamic acid, serine, glycine, alanine, and proline). The EAAI is a common index for estimating the quality of phytoplankton as a diet for higher trophic levels such as zooplankton (Oser, 1959; Mente et al., 2002; Becker and Richmond, 2004). The EAAI is defined as a ratio of EAA in prey to corresponding EAA in reference egg protein (Oser, 1959). However, the mean fraction of EAA in the zooplankton community in this study (unpublished data) was used as the reference AA since efficient food has a similar AA profile to that of the consumer (Guisande et al., 2002). The modified EAAI of POM was determined from this formula:

$$EAAI=\sqrt[{n}]{\frac{aa_{1}}{AA_{1}}\times \frac{aa_{2}}{AA_{2}}\times \frac{aa_{3}}{AA_{3}}\times \frac{aa_{4}}{AA_{4}}\times \cdots \times \frac{aa_{n}}{AA_{n}}}$$

where *aa*_1_, …, *aa*_*n*_ are the ratio of each EAA to total EAA in POM and *AA*_1_, …, *AA*_*n*_ are the average ratio of each EAA to total EAA in zooplankton communities in this study (unpublished data). To calculate EAAI, the values of *aa*_1_/*AA*_1_, …, *aa*_*n*_/*AA*_*n*_ were constrained between 0.01 minimally and 1 maximally (Hayashi et al., 1986).

### Statistical Analysis

Significant differences of biochemical properties (concentrations of biomolecules and biomolecular composition) between northern and southern stations were tested using the Student’s *t*-test. The results of statistical analyses were assumed to be significant at *p*-values < 0.05. All correlation analyses were performed in this study using Pearson’s correlation coefficients. Statistical analyses were performed using Statistical Package for the Social Sciences (SPSS ver.12.0). For the multivariate analysis of the analyzed and investigated parameters, we carried out the principal component analysis (PCA) with the rotation method of Varimax with Kaiser normalization using the XLSTAT software (Addinsoft, Boston, MA, United States). Further, to calculate the dissimilarity between samples, agglomerative hierarchical clustering (AHC) analysis with Euclidean distance dissimilarity and Ward’s method was conducted using the XLSTAT software (Addinsoft, Boston, MA, United States).

## Results

### Hydrographical and Chemical Properties

**FIGURE 2:**
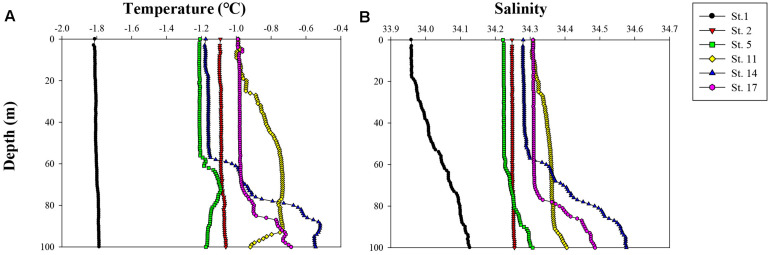
Vertical profiles of **(A)** temperature (°C) and **(B)** salinity in the upper 100 m of the water column during this cruise ANA08C.

The vertical profiles of potential temperature and salinity within the upper 100 m ranged from −1.82 to −0.52°C and from 33.96 to 34.58, respectively (Figure 2). The lowest potential temperature and salinity were measured at station (St.) 1, whereas other stations never reached freezing temperature and had relatively higher salinities. The higher salinity values over 34.40 with depth showed at Sts. 14 and 17 which also had higher temperature values. The *Z*_*eu*_ and *Z*_*m*_ were 30–43 and 20–117 m, respectively (Table 1). The *Z*_*eu*_ at most stations was shallower than *Z*_*m*_ except for Sts. 1 and 11.

**TABLE 1:** Description of sampling stations and associated environmental variables during ANA08C cruise.

Station	Latitude (°N)	Logitude (°E)	Date (mm/dd/yy)	T_*eu*_	S_*eu*_	Bottom depth (m)	Z_*eu*_ (m)	Z_*m*_ (m)
1	−72.318	170.177	25/02/18	−1.8	34.0	165	43	21
2	−71.698	172.186	26/02/18	−1.1	34.2	1043	35	117
5	−72.163	175.566	27/02/18	−1.2	34.2	1342	38	65
11	−72.987	174.315	28/02/18	−1.0	34.3	345	30	20
14	−72.596	171.413	28/02/18	−1.2	34.3	387	41	53
17	−73.421	173.662	01/03/18	−1.0	34.3	287	30	75

*T_eu_ and S_eu_: water temperature and salinity averaged from surface to the euphotic zone depth (Z_eu_).Z_m_: mixed layer depth.*

**FIGURE 3:**
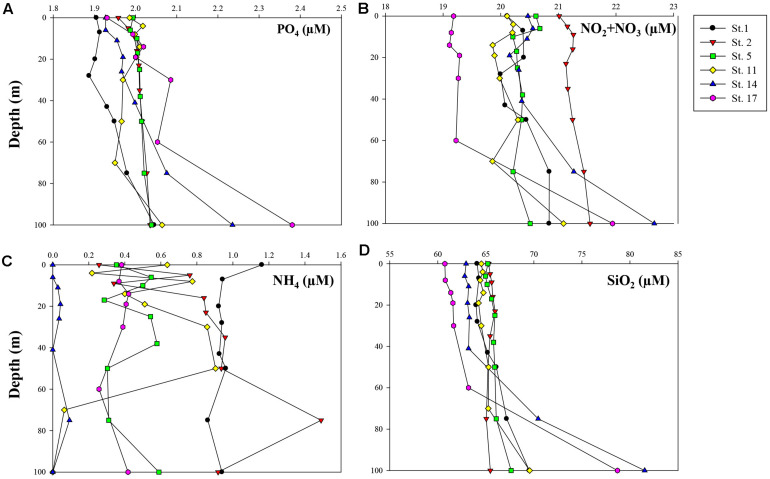
Vertical profiles of measured concentrations (μM) of major inorganic nutrients within 100 m depth: **(A)** phosphate (PO_4_), **(B)** nitrate + nitrite (NO_2_ + NO_3_), **(C)** ammonium (NH_4_), and **(D)** silicate (SiO_2_).

Figure 3 shows vertical distributions of dissolved inorganic nutrients (phosphate, nitrate + nitrite, ammonium, and silicate) from the surface to 100 m depth. The concentrations of dissolved inorganic nutrients except for ammonium mostly increased with depth from the surface to 100 m. At Sts. 14 and 17, the concentrations of phosphate, nitrate + nitrite, and silicate increased sharply below the euphotic layers compared with those at other stations. In the upper 100 m, the concentrations of phosphate, nitrate + nitrite, and silicate were 1.89–2.38, 19.11–22.66, and 60.74–81.53 μM, respectively. Dissolved inorganic ammonium had low concentrations, ranging from 0 to 1.49 μM, and did not show a clear spatial pattern.

### Particulate Organic Carbon (POC), Nitrogen (PON), and Stable Carbon Isotopes (δ^13^C) of POM

The averaged concentrations of POC, PON, and C/N ratio within the euphotic zone and δ^13^C values of surface POM are summarized in Table 2. The POC and PON concentrations were 108.0–194.5 and 11.1–27.5 μg L^–1^, respectively. The lowest mean values of the euphotic depth-averaged POC and PON were observed at St. 2 while the highest values were found at St. 17 (Table 2). The C/N ratios were in a range of 7.7–11.4 and the average C/N ratio value was highest at St. 1 (10.8 ± 0.6) and lowest at St. 17 (8.0 ± 0.3) (Table 2). The δ^13^C values of surface POM ranged from −25.1 (St. 1) to −29.2‰ (St. 17) (Table 2).

**TABLE 2:** The concentrations of POC and PON, and δ13C (‰) of particulate organic matter (POM) in the northwestern Ross Sea.

Station	Light depth (%)	Sampling depth (m)	POC (μg L^–1^)	PON (μg L^–1^)	C/N ratio (molar:molar)	POC/Chl-*a*	δ13C (‰)
1	100	0	166.4	19.0	10.2	299.7	−25.1
	30	11	147.4	15.1	11.4	262.9	
	1	43	128.3	13.8	10.9	225.4	
2	100	0	108.2	11.1	11.4	282.3	−26.3
	30	9	116.0	13.6	10.0	297.3	
	1	35	133.0	15.3	10.2	381.3	
5	100	0	125.0	15.7	9.3	343.2	−27.1
	30	10	127.4	15.2	9.8	354.0	
	1	38	108.0	14.3	8.9	295.4	
11	100	0	153.1	18.7	9.6	226.7	−26.9
	30	8	140.4	17.6	9.3	206.6	
	1	30	142.7	16.3	10.2	142.9	
14	100	0	141.7	17.6	9.4	192.1	−26.8
	30	11	133.5	16.6	9.4	188.6	
	1	41	144.4	17.5	9.6	173.7	
17	100	0	175.4	25.7	8.0	128.1	−29.2
	30	8	194.5	27.5	8.3	139.2	
	1	30	174.9	26.6	7.7	118.9	

### Phytoplankton Biomass and Community Structure

The vertical patterns of the total chl-*a* concentrations between the surface and 1% light depth are shown in Table 3 and were almost uniform throughout the euphotic zone at each station (Table 3). Depth-integrated total chl-*a* concentrations throughout the euphotic zone (from the surface to depth of Z_*eu*_) ranged between 13.1 and 42.6 mg chl-*a* m^–2^, with a mean value of 24.7 mg chl-*a* m^–2^ (SD = ±11.1 mg chl-*a* m^–2^). The lowest integrated chl-*a* values were observed at the northernmost Sts. 2 and 5, while the highest value was found at the southernmost and near the offshore station (St. 17). We found a distinct difference in chl-a contributions of different size classes (>20, 5–20, and 0.7–5 μm) to the total chl-*a* concentration among the stations (Table 3). The large-sized phytoplankton (>20 μm) contributed most to the total phytoplankton biomass in the southern part of the study area (Sts. 11, 14, and 17), whereas relatively smaller cells (0.7–5 and 5–20 μm) were dominating in the northern part (Sts. 1, 2, and 5). The overall contributions of large (>20 μm), middle (5–20 μm), and small-sized (0.7–5 μm) phytoplankton to the total chl-*a* concentrations were 14.7–82.8, 11.1–47.4, and 5.6–47.1%, respectively. Moreover, the contribution of large-sized fraction (>20 μm) showed a statistically significant positive correlation with integrated total chl-*a* concentration (*r* = 0.888, *p* < 0.05), whereas a significant negative correlation was found between small size-class (0.7–5 μm) and integrated total chl-*a* value (*r* = −0.910, *p* < 0.05) in our study area.

**TABLE 3:** Total and euphotic-depth integrated chl-*a* concentrations (from the surface to 1% light depth) and compositions of different size-fractionated chl-*a* and phytoplankton communities.

Station	Light depth (%)	Total		Size-fractionated (%)			Phytoplankton community composition (%)			
		Chl-*a* (μg L^–^^1^)	Integrated (mg chl-*a* m^–^^2^)	>20 μm	5–20 μm	0.7–5 μm	Diatoms	Haptophytes	Dinoflagellates	Others
1	100	0.6	24.2	16.6	40.0	43.4	74.3	25.2	0.3	0.2
	30	0.6		29.0	43.1	27.9	76.4	23.1	0.3	0.2
	1	0.6		25.4	47.4	27.2	77.5	22.0	0.4	0.0
2	100	0.4	13.1	17.1	39.2	43.7	37.1	62.4	0.3	0.2
	30	0.4		16.2	36.7	47.1	25.3	74.4	0.4	0.0
	1	0.3		14.7	42.8	42.5	33.9	65.7	0.4	0.0
5	100	0.4	13.8	15.3	43.5	41.2	48.7	48.1	0.0	3.2
	30	0.4		16.5	43.2	40.3	54.6	42.0	0.0	3.4
	1	0.4		18.8	42.4	38.8	55.2	41.0	0.0	3.8
11	100	0.7	23.9	59.4	22.0	18.7	72.2	20.8	0.6	6.4
	30	0.7		60.7	22.3	17.0	79.6	16.1	0.5	3.8
	1	1.0		71.5	18.6	9.9	91.4	5.6	0.6	2.5
14	100	0.7	30.7	59.2	25.9	14.9	93.1	0.2	0.6	6.1
	30	0.7		64.4	21.4	14.3	79.1	18.9	0.6	1.4
	1	0.8		50.4	35.0	14.6	74.6	22.3	0.9	2.2
17	100	1.4	42.6	82.5	11.9	5.6	95.5	0.1	0.3	4.1
	30	1.4		82.5	11.1	6.3	95.1	0.0	0.6	4.3
	1	1.5		82.8	11.3	5.9	95.8	0.1	0.3	3.7

Concerning the relative contributions of individual phytoplankton groups based on CHEMTAX analysis (Mackey et al., 1996; Wright et al., 1996; DiTullio et al., 2011), the combined contributions of diatoms and haptophytes (hereinafter *Phaeocystisantarctica*) contributed up to 99.6% of the total phytoplankton biomass during this study (Table 3). The phytoplankton community composition exhibited varying vertical distribution patterns and did not show clear differences between the three light depths (Table 3). Although most of the stations were diatoms-dominated with relatively low contributions of *P. antarctica*, *P. antarctica* presented markedly higher contributions at Sts. 2 and 5 (mean ± SD = 67.5 ± 6.2 and 43.7 ± 3.8%, respectively) (Table 3). Since two major groups, diatoms and *P. antarctica*, were distinctly observed during our study period, they could be related with different cell sizes based on our size-fractionated chl-*a* results. The size-fractionated chl-*a* and pigment analyses revealed that the larger phytoplankton assemblage (>5 μm) was dominated by diatoms (*r* = 0.917, *p* < 0.05). In comparison, the greater fraction of chl-*a* contained in small cells (<5 μm) accounted for mainly *P. antarctica* (*r* = 0.929, *p* < 0.01), presumably indicative for solitary cells of *P. antarctica* (∼4 μm in size; Schoemann et al., 2005).

### Biomolecular Composition of POM

The concentrations and relative composition of CHO, PRT, LIP, and food material (FM; the sum of CHO, PRT, and LIP; Danovaro et al., 2000) concentrations are presented in Table 4. The differences in concentrations of each biochemical pool (CHO, PRT, and LIP) and FM at these different three-light depths did not show a clear pattern. The absolute concentrations of each biomolecule (CHO, PRT, and LIP) and FM were 67.4–161.6, 7.9–75.6, 43.8–118.0, and 163.4–278.4 μg L^–1^, respectively (Table 4). There was no clear difference in the CHO concentrations among the stations. In contrast, the PRT, LIP, and FM contents exhibited higher values in the southern part compared to those in the northern part (*t*-test, *p* < 0.05), in particular for PRT and FM contents (*t*-test, *p* < 0.001). Based on Pearson’s correlation analysis, the PRT, LIP, and FM contents were found to have a positive relationship with chl-*a* concentration as an indicator of phytoplankton biomass (PRT vs. Chl-*a*: *r* = 0.915, *p* < 0.01; LIP vs. Chl-*a: r* = 0.787, *p* < 0.01; FM vs. Chl-*a*: *r* = 0.806, *p* < 0.01). Regarding the relative percentages of biomolecular components at each station, CHO made up the largest portion with a mean percentage of 54.0 ± 10.2%, increasing to ∼66.7% at the 1% light depth, followed by LIP (mean ± SD = 29.9 ± 5.4%) and PRT (mean ± SD = 16.1 ± 6.8%) (Table 4). Although the biomolecular compositions of phytoplankton varied without vertical trends, the CHO and PRT compositions exhibited spatial variability between the northern and southern parts (Table 4). More specifically, CHO compositions in the northern part (Sts. 1, 2, and 5) were higher than those in the southern part (Sts. 11, 14, and 17) (*t*-test, *p* < 0.01). In comparison, PRT composition in the euphotic layer of the southern part has much higher values compared to those measured in the northern part (*t*-test, *p* < 0.001). Meanwhile, we found that the proportion of CHO positively correlated with *P. antactica* composition (*r* = 0.609, *p* < 0.01) while the proportion of PRT positively correlated with diatom composition (*r* = 0.592, *p* < 0.01).

**TABLE 4:** Concentrations of each biomolecular component (CHO, PRT, and LIP) and food materials (FMs) and percentages of the biomolecular composition of POM at each station in the northwestern Ross Sea.

Station	Light depth (%)	Concentration (μg L^–1^)				Composition (%)		
			
		CHO	PRT	LIP	FM	CHO	PRT	LIP
1	100	122.8	23.1	53.3	199.2	61.6	11.6	26.8
	30	118.4	7.9	58.4	184.7	64.1	4.3	31.6
	1	121.3	11.4	49.3	182.0	66.7	6.2	27.1
2	100	122.8	22.4	50.7	195.9	62.7	11.4	25.9
	30	97.8	23.1	43.8	164.7	59.4	14.0	26.6
	1	114.8	16.9	47.4	179.1	64.1	9.4	26.5
5	100	96.8	21.7	44.9	163.4	59.2	13.3	27.5
	30	102.4	22.4	70.5	195.3	52.4	11.5	36.1
	1	84.4	23.1	58.4	166.0	50.9	13.9	35.2
11	100	125.7	42.5	54.4	222.5	56.5	19.1	24.4
	30	161.6	36.9	62.1	260.6	62.0	14.2	23.8
	1	97.1	42.5	64.3	203.8	47.6	20.8	31.5
14	100	86.9	37.6	62.8	187.3	46.4	20.1	33.5
	30	109.4	43.1	56.9	209.5	52.2	20.6	27.2
	1	121.3	41.8	61.0	224.1	54.1	18.6	27.2
17	100	100.5	61.1	73.8	235.3	42.7	26.0	31.3
	30	67.4	69.4	118.0	254.9	26.5	27.2	46.3
	1	118.4	75.6	84.4	278.4	42.5	27.2	30.3

### Amino Acid Composition of POM and Amino Acid Indices

Particulate hydrolyzable AA concentrations were quantified from the sum of each measured concentration of 14 detected AA since tyrosine, cystine, and tryptophan were not detected in our POM samples (Table 5). The PAA concentrations within the euphotic zone ranged from 0.18 μM at the 30% light depth of St. 2 to 1.04 μM at the 30% light depth of St. 17, with an average value of 0.40 ± 0.21 μM (Table 5). However, the significant spatial distinction of PAA concentrations was not observed although the concentrations of biomolecular components had a clear spatial pattern as mentioned above. We calculated the carbon and nitrogen normalized yields of AAs (AA-POC% and AA-PON%) for our PAA samples (Table 5). The PAA accounted for 7.8–26.6% of total POC (mean ± SD = 14.2 ± 5.9%) and 21.3–81.8% of total PON (mean ± SD = 41.7 ± 19.7%), respectively (Table 5).

**TABLE 5:** PAA concentrations, carbon and nitrogen normalized yields of PAA (AA-POC% and AA-PON%), mol fractions of individual amino acids, NEAA, and EAA, and values of AA-based indices (DI and EAAI).

Station	light depth (%)	PAA (μM)	AA- POC%	AA- PON%	Amino acid composition (mol%)														NEAA (mol%)	EAA (mol%)	DI	EAAI
									
					ASP	GLU	SER	GLY	ALA	HIS	THR	ARG	VAL	MET	PHE	ILE	LEU	LYS				
1	100	0.30	9.6	28.9	6.40	14.48	7.67	17.98	8.83	3.59	7.69	6.14	5.94	1.51	3.92	4.31	7.62	3.94	55.4	44.6	−0.10	0.87
	30	0.61	21.3	76.9	6.61	18.01	7.64	22.86	8.98	4.03	3.00	8.18	3.77	2.48	2.54	3.50	7.24	5.52	64.1	40.3	−0.72	0.83
	1	0.61	24.0	81.8	4.65	10.33	9.45	25.39	6.87	4.04	7.23	7.67	4.13	4.33	3.59	3.62	8.68	N.D.	56.7	43.3	−0.03	0.56
2	100	0.22	10.2	36.6	6.64	10.75	7.69	22.88	11.26	4.96	6.02	7.35	1.55	6.30	4.01	3.36	7.90	N.D.	59.2	41.5	−0.11	0.51
	30	0.18	7.8	23.6	6.31	10.06	7.84	20.74	13.30	6.04	4.30	5.82	1.72	7.16	4.32	4.28	8.11	N.D.	58.2	41.8	0.84	0.51
	1	0.37	13.8	44.2	5.42	13.28	7.82	24.80	9.75	4.67	4.68	7.49	1.39	6.02	3.36	3.42	7.28	N.D.	61.1	38.3	−0.02	0.51
5	100	0.21	8.2	21.3	9.65	11.71	8.68	24.90	10.38	N.D.	5.27	4.71	6.93	1.88	4.31	4.25	7.33	N.D.	65.3	34.7	−0.54	0.34
	30	0.46	18.8	59.1	7.57	15.38	6.71	23.69	7.65	5.49	4.11	7.04	1.26	4.90	4.00	3.04	3.33	9.68	61.0	42.8	−0.04	0.71
	1	0.43	20.7	56.3	7.63	20.28	6.05	22.23	7.62	6.59	3.44	7.06	2.31	1.50	3.87	3.23	8.18	N.D.	63.8	36.2	0.00	0.49
11	100	0.29	9.9	28.4	5.35	10.84	7.53	21.56	11.20	3.71	7.25	7.11	3.92	3.87	4.25	4.26	9.16	N.D.	56.5	43.5	0.29	0.57
	30	0.26	10.4	27.1	10.01	13.38	8.14	14.97	N.D.	3.60	8.60	6.10	4.28	4.71	4.76	5.99	9.33	6.11	46.5	53.5	1.00	0.94
	1	0.51	18.5	56.7	6.16	10.58	7.56	19.91	11.05	2.67	7.48	6.62	3.63	3.85	4.05	4.57	8.21	3.65	55.3	44.7	0.03	0.92
14	100	0.25	9.5	23.8	9.83	12.79	7.26	11.96	11.75	N.D.	7.51	4.64	4.46	5.00	4.27	6.18	8.39	6.44	53.6	46.9	0.45	0.57
	30	0.25	10.0	27.9	6.67	11.27	7.53	18.04	10.71	4.23	6.75	5.72	4.75	3.39	3.92	4.85	7.91	4.26	54.2	45.8	0.24	0.95
	1	0.29	10.5	28.3	7.17	12.84	7.96	16.48	11.05	N.D.	7.72	6.06	6.68	2.37	3.91	4.93	8.18	4.64	55.5	44.5	−0.18	0.57
17	100	0.41	12.0	29.0	6.19	9.82	7.44	22.44	10.31	3.55	7.35	6.34	4.36	3.23	3.80	4.50	7.92	2.73	56.2	43.8	−0.16	0.90
	30	1.04	26.6	68.6	6.39	12.83	6.71	23.91	9.80	4.42	7.12	6.37	5.57	1.75	2.38	3.14	7.88	0.64	59.6	39.3	−0.87	0.71
	1	0.47	13.7	32.9	5.98	8.91	6.88	23.09	9.85	5.78	7.20	6.22	5.48	1.99	3.40	4.04	8.71	2.48	54.7	45.3	−0.07	0.84
Mean ± SD		0.40 ± 0.21	14.2 ± 5.9	41.7 ± 19.7	6.92 ± 1.52	12.64 ± 2.94	7.59 ± 0.76	20.99 ± 3.74	9.46 ± 2.85	3.74 ± 1.99	6.26 ± 1.68	6.48 ± 0.95	4.01 ± 1.78	3.68 ± 1.75	3.85 ± 0.60	4.19 ± 0.91	7.85 ± 1.27	2.78 ± 2.93	57.6 ± 4.5	42.8 ± 4.3	0.00 ± 0.47	0.68 ± 0.19

*The list of AA abbreviations is as follows: aspartic acid (ASP), glutamic acid (GLU), serine (SER), glycine (GLY), alanine (ALA), histidine (HIS), threonine (THR), arginine (ARG), valine (VAL), methionine (MET), phenylalanine (PHE), isoleucine (ILE), leucine (LEU), and lysine (LYS).*

The contributions of each AA in the PAA are presented in Table 5. The AA composition in the PAA was variable among the stations, and especially lysine and histidine have substantial variability. The major constituents of the PAA were glycine, glutamic acid, and alanine whereas lysine, histidine, methionine, and phenylalanine were minor components (Table 5). Among individual AA, glycine was the most dominant constituent (mean ± SD = 20.99 ± 3.74%), followed by glutamic acid, alanine, leucine, and serine. Lysine had the lowest molar percentage (mean ± SD = 2.78 ± 2.93%) and particularly was below the detection limit in most samples obtained from Sts. 2 and 5. The percentage compositions of NEAA and EAA to total PAA were in the ranges of 46.5–65.3 and 34.7–53.5%, respectively (Table 5). The proportion of NEAA was higher than those of the EAA fraction except for the sample at the 30% light depth of St. 11.

**FIGURE 4:**
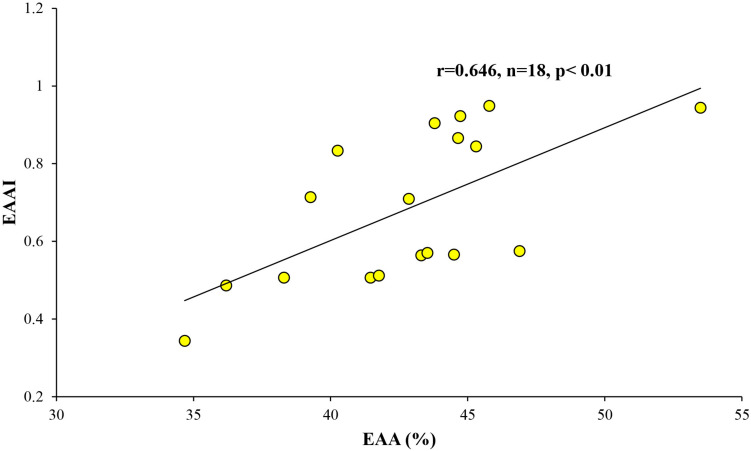
Relationship between the relative contribution of EAA (%) and EAAI.

The calculated DI and EAAI values ranged from −0.87 to 1.00 and from 0.34 to 0.95, respectively (Table 5). In this study, strong positive correlation was found between the relative contribution of EAA (%) and EAAI (Figure 4; *r* = 0.629, *p* < 0.01).

### Multivariate Analysis (AHC Analysis and PCA) Between Biochemical (Biomolecular and Amino Acid) Compositions of POM and Other Chemical and Biological Features

**FIGURE 5:**
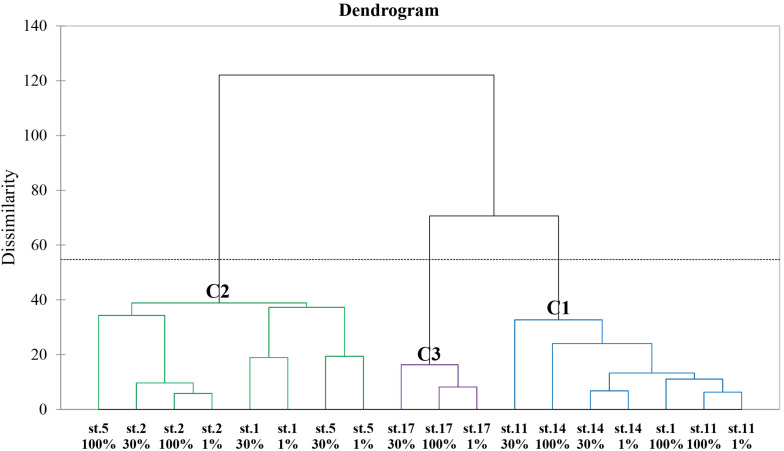
Dendrogram representing the agglomerative hierarchical clustering (AHC) based on dissimilarities using Ward’s method between the biochemical (biomolecular and amino acid) compositions of POM and other 11 chemical and biological features.

The dendrogram according to AHC analysis based on the same 28 variables used with PCA revealed three distinct groups (Figure 5). Cluster 1 (C1) had the southern part samples and one sample collected from 100% light depth at St. 1 whereas Cluster 2 (C2) contained almost all the samples obtained from the northern section. Cluster 3 (C3) included the samples collected from only St. 17 in the southern part. The maximum value of distances between the class centroids was observed between C2 and C3 (29.743), and C1 and C3 had a small difference in dissimilarity.

**FIGURE 6:**
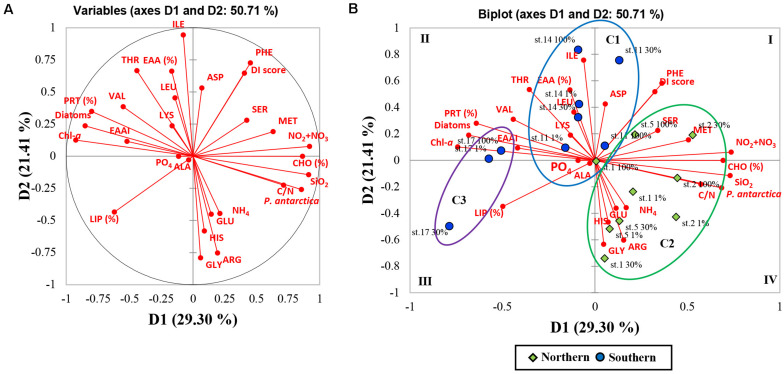
Principal component analysis (PCA; after normalized varimax rotation) based on the biochemical (biomolecular and amino acid) compositions of POM and other chemical and biological features. **(A)** Correlation circle and projection obtained by PCA of the 28 variables; each red arrow represents the squared cosine corresponding to an individual variable. **(B)** Biplot of PCA on the 28 variables and observations for the first and second axis (variability explained: 50.71%). Observations are grouped according to the classes obtained from agglomerative hierarchical clustering (AHC). Each abbreviation represents as aspartic acid (ASP), glutamic acid (GLU), serine (SER), glycine (GLY), alanine (ALA), histidine (HIS), threonine (THR), arginine (ARG), valine (VAL), methionine (MET), phenylalanine (PHE), isoleucine (ILE), leucine (LEU), lysine (LYS), compositions of essential amino acids (EAAs), essential amino acid index (EAAI), degradation index (DI), relative contributions of diatoms (Diatoms) and haptophytes (*P. antarctica*), biomolecular composition (CHO, PRT, and LIP), carbon to nitrogen ratio (C:N), phosphate (PO_4_), nitrate + nitrite (NO_2_ + NO_3_), ammonium (NH_4_), silicate (SiO_2_), and chlorophyll a (Chl-*a*).

As a result of the PCA, the principal components (PC) 1 and 2 explained 29.30 and 21.41% of the data variance in the biochemical compositions and other parameters among the stations (Figure 6). The PC1 was positively correlated with NO_2_ + NO_3_, SiO_2_, C/N ratio, CHO composition (%), *P. antarctica*, methionine, and serine whereas negatively loaded with Chl-a, PRT and LIP composition (%), Diatoms, EAAI, and valine. The PC2 was found to be positively loaded with EAA composition (%), isoleucine, threonine, phenylalanine, leucine, aspartic acid, and DI score while had negative loadings for glycine, arginine, and histidine. To determine whether correlations were worthy of interpretation, we examined the squared cosines of the variables and then excluded low values of squared cosines between the variables and PCs (Figure 6; i.e., glutamic acid, alanine, lysine, PO_4_, and NH_4_). A distinct spatial separation between the northern and southern parts of our study area was founded along the PC1 axis. In other words, most of the samples collected from the southern part were on the left side of the biplot (quadrants II and III) whereas most of the northern part samples were placed on the lower right-hand side (quadrant IV). Moreover, the observations in the PCA space showed a similar pattern of clustering as the AHC analysis (Figures 5, 6).

## Discussion

### Source of the Bulk POM

The marine POM filtered on filter paper includes diverse organic matter derived from phytoplankton, bacterial plankton, detritus, and terrestrial organic matter (Harmelin-Vivien et al., 2008). Both of C/N molar ratio and δ^13^C value in bulk POM have long been used as indicators of the nature of the organic matter in various marine ecosystems (Wada et al., 1975; Zweifel et al., 1993; Montagnes et al., 1994; Lee and Whitledge, 2005). Generally, phytoplankton have higher C/N ratios in a range of 6–10 than bacteria (3–5), whereas terrestrial organic matters have 2–20 times higher than the C/N values of phytoplankton (Brzezinski, 1985; Montagnes et al., 1994; Tyson, 1995; Goñi et al., 2003; Lamb et al., 2006). The range of C/N molar ratios of 7.7–11.4 (mean ± S.D = 9.6 ± 1.0) for POM in this study is within the previously reported range of C/N ratio for phytoplankton (Table 2). However, Fabiano et al. (1993) reported lower C/N molar ratios within the euphotic zone than our observation, ranging from 5.4 and 9.1 at the stations near Cape Adare and 5.4–6.6 at the stations located in Terra Nova Bay, Ross Sea. Moreover, Coale et al. (2003) obtained slightly lower ratios ranging from 6.5 to 7.9 in the southern Ross Sea. Generally, lower C/N ratios within the euphotic layer were reported during the bloom period in Antarctic water according to previous studies (Bodungen et al., 1986; Nelson et al., 1989; Fabiano et al., 1993). The relatively higher values of the C/N ratio in this study could be due to our sampling period conducted at the end of February 2018 in a post-bloom period which will be discussed later.

In general, δ^13^C values derived from marine phytoplankton range from −23 to −19‰ (Fry and Sherr, 1989; Harmelin-Vivien et al., 2008). In comparison, δ^13^C values in phytoplankton communities of Antarctic surface waters are lower than those observed in lower-latitude oceans (Wada et al., 1987; Rau et al., 1989, 1991; Dehairs et al., 1997). In this study, the δ^13^C values in the surface bulk POM were in a range of −29.2 to −25.1‰ (mean ± SD = −26.9 ± 1.3‰) (Table 2). Although our study has δ^13^C values closer to terrestrial organic matters (−30 to −26‰; Fry and Sherr, 1989), previous studies have reported that input of terrestrial organic matter from the ice-covered continent is negligible in the Ross Sea (Rogers and Dunbar, 1993; Villinski et al., 2000). Based on the results of both C/N ration and δ^13^C values of POM, the main source of POM in this study could be phytoplankton-derived organic matter.

### Biomolecular Composition of POM

In general, the temporal dynamics of the two major types of phytoplankton blooms have been well documented in the Ross Sea (Arrigo et al., 1999; Smith et al., 2000, 2011). The initial bloom dominated by the haptophyte *P. antarctica* is commonly found in the south-central Ross Sea during austral spring (Peloquin and Smith, 2007). The following second bloom dominated by diatoms commonly is observed in the western and eastern portions of the Ross Sea in summer (DiTullio and Smith, 1996; Peloquin and Smith, 2007). Considering temporal and spatial phytoplankton bloom patterns in the northwestern Ross Sea, we infer that our sampling period (end of February–the beginning of March) was in the post-bloom. In the present study, haptophytes dominated by solitary *P. antarctica* cells and nano-sized diatom assemblages with low chl-*a* concentrations (<0.6 μg L^–1^) and high POC/Chl-*a* values potentially indicate mostly inactive cells (Mangoni et al., 2017) prevailed in the northern part of the study area (Tables 2, 3). Smith et al. (2003) reported that the abundance of solitary *P. antarctica* cells increases in the Ross Sea during late summer under inorganic nutrient and/or iron limitation. Furthermore, Mathot et al. (2000) and Shields and Smith (2009) suggested that colonial *P. antarctica* cells can be associated with their maximum biomass under nutrient-replete conditions and exponential phase whereas solitary *P. antarctica* cells numerically dominate when their growth rate declined and the senescence phase began after bloom. In this study, the micro-sized diatoms with their elevated chl-*a* concentrations (up to 1.5 μg L^–1^) and low POC/Chl-*a* values indicate relatively active cells (Mangoni et al., 2017) predominated in the southern part (Tables 2, 3) in comparison to the northern part. Therefore, taking into account the bloom phase and biological features (phytoplankton community, cell size, chl-*a* concentration, and POC/Chl-*a* value), it seems reasonable to suggest that solitary *P. antarctica* cells and nano-sized diatoms observed in the northern section were in a senescent status, while micro-sized diatoms dominated in the southern section were in a relatively active condition.

There have been numerous studies published in the literature that the synthesis of biomolecular classes could be influenced by different growth phases of phytoplankton (Moal et al., 1987; Fernández-Reiriz et al., 1989; de Madariaga, 1992; Ríos et al., 1998; Ahn et al., 2019). Considerable changes in the biomolecular composition of the phytoplankton occurred throughout different growth phases (i.e., exponential, stationary, and senescent phases) (Ahn et al., 2019 and the references therein). The amounts of PRT as biomolecular products of photosynthesis increased during the exponential growth phase, indicating a higher PRT demand for exponential cell division and growth (Mayzaud et al., 1990; Berdalet et al., 1994). When phytoplankton become stationary and senescent conditions concurrent with the nutrient deficiency thereafter, CHO and LIP levels increase for energy reserves (Myklestad, 1974; Mague et al., 1980; Barlow, 1982; Tonon et al., 2002). Generally, phytoplankton growth in the Ross Sea is limited by irradiance during austral spring (Smith et al., 2000; Peloquin and Smith, 2007), but by nutrient bioavailability (particularly iron) in austral summer (Sedwick and Ditullio, 1997; Sedwick et al., 2000; Peloquin and Smith, 2007). In this study, major inorganic nutrient concentrations (phosphate, nitrate + nitrite, ammonium, and silicate) in seawater were not depleted (Figure 3). Furthermore, Si^∗^ (defined as [Si]- [NO_3_^–^] in μM) for the identification of potential iron limitation had positive values from all stations, suggesting that there was no evidence for iron limitation during this study (Sarmiento et al., 2004; Le Moigne et al., 2013). However, scarcity of iron is a very common feature in the Ross Sea during the summer season (Olson et al., 2000; Smith and Asper, 2001), and Si^∗^ could be restricted as a community-wide iron limitation index because Si^∗^ represents the silicic acid uptake and growth related to only diatom communities under iron limitation (Hogle et al., 2018; Louropoulou et al., 2019). On the other hand, the specific carbon uptake rates of phytoplankton in parallel with our study were highest at surface water from all stations, which suggests potential light limited conditions in late austral summer period (Lee et al., 2008). Hence, phytoplankton during this study could have been a physiologically inactive condition under unfavorable environmental conditions.

In our study, CHO accounted for the highest portion (mean ± SD = 54.0 ± 10.2%) among different biomolecules (CHO, PRT, and LIP) of POM (Table 4). However, the higher contribution of PRT (up to 27.2%) and lower contribution of CHO were observed in the southern section of our study area compared to those in the northern section (Table 4). Furthermore, we found significant differences in CHO and PRT compositions between the northern and southern stations (*t*-test, *p* < 0.05). These discrepancies could explain that CHO-rich solitary *P. antarctica* cells and nano-sized diatoms were in a senescent phase in the northern part whereas micro-sized diatoms having relatively higher PRT had more active conditions in the southern part. On the other hand, marked spatial variations in biomolecular compositions among the stations were probably due to taxonomic differences. The composition of CHO-rich POM may be linked with structural and/or storage CHO synthesis of *P. antarctica* (Lancelot and Mathot, 1985; Alderkamp et al., 2007; Mangoni et al., 2017; Kim et al., 2018). *P. antarctica* produces a mucous colony matrix which is mostly composed of polysaccharides as a kind of structural CHO (Alderkamp et al., 2007; Mangoni et al., 2017). Hong et al. (1997) suggested that when *P. antarctica* colony matrix begins to break up during the senescent phase, transparent exopolymer particle (TEP) production by *P. antarctica* is closely related with increased particulate CHO. Moreover, CHO accumulation has been observed when both single-cell and colonial *P. antarctica* reach the end of the bloom phase since they store the surplus energy as storage CHO (Lancelot and Mathot, 1985; Alderkamp et al., 2007 and the references therein). In contrast, Young et al. (2015a) found that Antarctic diatoms adapted to cold temperatures tend to increase PRT concentrations to compensate for slow enzyme rates. In conclusion, spatial variability of the biomolecular composition in the bulk POM during this study was not only influenced by phytoplankton growth phases but also by those taxonomic compositions.

### Influence of Origin and Degradation Status on the Amino Acid Composition of POM

The measured concentrations of the PAA during this study (Table 5; 0.18–1.04 μM) varied significantly but were in agreement well with the range of values previously reported from polar regions (Hubberten et al., 1995; Dittmar et al., 2001; Tsukasaki and Tanoue, 2010; Tremblay et al., 2015). Based on the Antarctic data (Weddell Sea), Hubberten et al. (1995) found relatively higher PAA concentrations (0.75 ± 0.60 μM) averaged in the upper 100 m depth than those reported in the Arctic water (mean ± SD = 0.57 ± 0.61 μM). Tremblay et al. (2015) observed higher concentrations of PAA at the most productive stations (up to 0.82 μM) while lower concentrations of PAA (0.16–0.22 μM) at the stations with low phytoplankton biomass in the Southern Ocean. Our results are also consistent with that the positive relationship between PAA and total chl-*a* concentrations (*r* = 0.510, *p* < 0.05). Therefore, the source of PAA in this study is probably mostly phytoplankton-produced PRT (Kalachova et al., 2004 and the references therein).

It is well known that AAs in hydrolyzed POM accounted for approximately 30% of POC and 50% of PON in various oceans (Handa, 1970; Siezen and Mague, 1978; Liebezeit and Bölter, 1986; Misic et al., 2017). All of the AA contributions to POC (Table 5; 7.8–26.6% of total POC) in the present study are lower than those in previous studies (Handa, 1970; Siezen and Mague, 1978; Liebezeit and Bölter, 1986). In contrast, the averaged proportions of AA to PON (mean ± SD = 41.7 ± 19.7% of total PON) are comparable to those in previous studies (Handa, 1970; Siezen and Mague, 1978; Liebezeit and Bölter, 1986), although they varied greatly (21.3–81.8% of total PON) (Table 5). According to Shields et al. (2019), the carbon normalized yield of AAs (AA-POC%) had higher values in less degraded organic matter and decreased with degradation. In other words, values of AA-POC% were highest during the mid-exponential bloom phase, while they decreased in the stationary and degradation phases of phytoplankton growth (Shields et al., 2019). Furthermore, AA-PON% could also be indicated for diagenesis in phytoplankton (Duan and Bianchi, 2007). Therefore, the relatively low AA contributions to the total POC and PON in this study imply that the majority of PAA might have undergone degradation to some degree (Duan and Bianchi, 2007; Shields et al., 2019).

The major constituents of PAA during this cruise were glycine, glutamic acid, and alanine, occupying 43.1% (±5.1%) of total PAA in the bulk POM (Table 5). Generally, previous studies reported that the predominant AAs of phytoplankton are glutamic acid, aspartic acid, alanine, and leucine regardless of marine or freshwater species although there are little differences in the AA composition of phytoplankton depending on the species (Hayashi et al., 1986 and the references therein). However, Hecky et al. (1973) suggested that serine + threonine and glycine could be enriched in the cell wall PRT of diatoms. We found that mol% serine + threonine only positively correlated with diatom composition (*r* = 0.473, *p* < 0.05) while mol% glycine had no correlation with diatoms. Although the correlation directly with diatoms was poor as diatom frustules can be preferentially preserved after cell death, glycine and serine were found to be bounded on the diatom frustules and this may be a reason why glycine was enriched in the POM (Ingalls et al., 2006). On the other hand, Liebezeit and Bölter (1986) found that glutamic acid, aspartic acid, glycine, and serine are the most dominant compounds of phytoplankton-derived PAA whereas glycine becomes dominant in the PAA of deeper waters with an appreciable quantity of detrital materials. Thus, the composition of PAA was caused by the combined effects of diatom-dominated phytoplankton communities and phytodetritus in this study after the bloom. Further evidence for supporting the degraded POM in our study was relatively low DI values of PAA (Table 5). Over half of calculated DI scores for our PAA samples showed negative values indicating that PAA appeared to be highly degraded phytodetritus (Dauwe et al., 1999; Wu et al., 2007; Shields et al., 2019). In general, the DI scores can provide information on the degree of degradation in bulk POM (Dauwe et al., 1999; Wu et al., 2007; Shields et al., 2019). The more negative DI value indicates the more degraded condition, while a positive DI value is indicative of fresh phytoplankton (Dauwe et al., 1999; Wu et al., 2007; Shields et al., 2019).

### The Potential Impacts of the AA Composition on Food Quality for Zooplankton Nutrition

The nutritional quality of PRT can be estimated by the proportion of total EAA and EAAI (Mente et al., 2002; Ju et al., 2008). As shown in Table 5, total EAA contributed 42.8 ± 4.3% during this study, which is within the range (41–55%) of compositional data on the EAA of microalgae and cyanobacteria conducted both in laboratory cultures and natural conditions (Kolmakova and Kolmakov, 2019 and the references therein). The general profile for individual EAA of phytoplankton composed high contributions of leucine and arginine whereas methionine and histidine were significantly lower than other EAA (Kolmakova and Kolmakov, 2019 and the references therein). In this study, however, lysine and histidine were limited in our POM samples collected from some stations with concurrent lower values of the EAAI (Table 5). EAAI scores can be evaluated for protein quality by comparing the geometric mean value of EAA in an FM relative to a reference protein derived from consumers (Peñaflorida, 1989). Based on the classification of Oser (1959), scores of the calculated EAAI over 0.9 are defined as good protein material, EAAI of approximately 0.8 is indicated as a useful protein, and EAAI below 0.7 can be classified as inadequate PRT. Thus, efficient protein food can be considered by the most similar AA profile between prey and their consumer and EAAI scores approaching 1.0 (Ju et al., 2008). The mean EAAI (0.68 ± 0.19) was classified as inadequate protein FMs during this study although the scores of the total EAAI (0.34–0.95) varied significantly (Table 5). Based on the results of EAA in this study, we found that significant positive relationship between the proportion of EAA and AA EAAI (Figure 4). This result may be surmised that a greater proportion of EAA in POM was composed with EAA composition with balanced in an optimal proportion.

**FIGURE 7:**
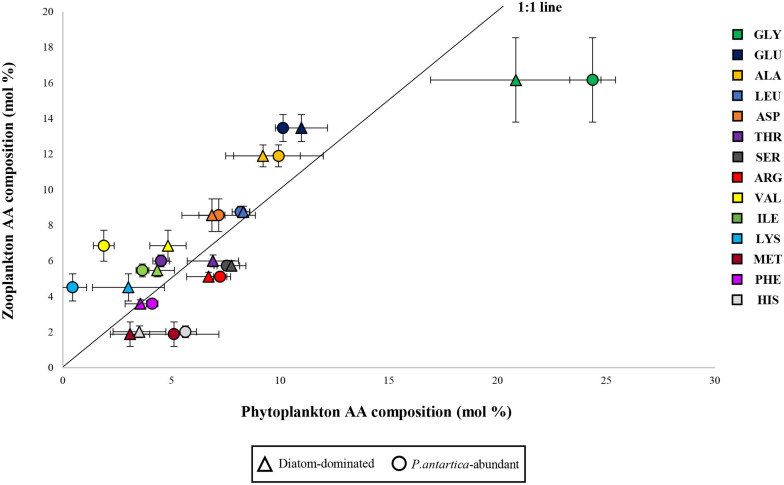
Relationship between amino acid profiles of diatom-dominant and *P. antartica-*abundant communities and those in zooplankton communities as potential consumers. Each abbreviation represents aspartic acid (ASP), glutamic acid (GLU), serine (SER), glycine (GLY), alanine (ALA), histidine (HIS), threonine (THR), arginine (ARG), valine (VAL), methionine (MET), phenylalanine (PHE), isoleucine (ILE), leucine (LEU), and lysine (LYS).

Anderson et al. (2004) suggested that individual EAA-deficient diets had a greater impact on the limitation of the growth of higher trophic levels rather than bulk amounts of protein and nitrogen. The previously published studies found that imbalances in dietary EAA could cause a bad influence on the growth of marine zooplankton (Kleppel et al., 1998; Guisande et al., 2000). Furthermore, the total AA composition of the copepod diets, as well as EAA composition, can be important for the higher reproductive success of copepods (Guisande et al., 2002). According to Guisande et al. (2000), AA from ingested food could not be converted into consumer’s biomass for egg production with an optimal proportion of AA if AA composition in prey is highly dissimilar to that of female copepods. Thus, the higher reproductive success of female copepods is observed when the AA composition of the ingested food is similar to that of the consumers (Guisande et al., 1999, 2000). In this study, we compared the averaged each AA profile of two phytoplankton communities that were divided into diatoms-dominant (Sts. 1, 11, 14, and 17) and relatively higher *P. antarctica-*abundant communities (Sts. 2 and 5) with those of bulk zooplankton communities (unpublished data) (Figure 7). The reason why we separated into two groups is that variable grazing by herbivores appears to discriminate based on the food quality, preference of ingesting cells, and distributions of the phytoplankton community (Haberman et al., 2003 and the references therein). In addition, lysine was nearly absent throughout the euphotic zone at *P. antarctica*-abundant stations 2 and 5 (Table 5). Assuming that the same assimilation rates of total AA between the two groups, we observed that the relationship for the diatoms-dominant group was closer to the 1:1 line considered as the ideal line in comparison to *P. antarctica-*abundant group with greater deviations of glycine, lysine, valine, methionine, and histidine from the line (Figure 7). Therefore, it seems reasonable to suggest that diatoms-dominant diets were better protein sources because they had an AA composition similar to their consumers and higher EAAI value. Our findings are also consistent with the conclusions of Boyd (1989) and Burford (1997).

### The Application of Multivariate Statistical Analysis for Evaluating Food Quality for Consumers

In this work, the multivariate statistical analysis was conducted for finding relationships between biochemical compositions (biomolecular and AA compositions) and other chemical and biological data. The PC1 of PCA results separated two different groups of biochemical parameters and phytoplankton communities. These two groups formed high proportions of CHO and high C/N values with a haptophytes-abundant group dominated by solitary *P. antarctica* cells while relatively higher proportions of PRT and high EAAI scores with diatom-dominant communities (Figure 6). In the PC2, EAA composition and DI score were negatively related to glycine which is considered as an indicator of organic matter degradation as discussed above (Figure 6) (Liebezeit and Bölter, 1986; Petersson and Floderus, 2001). Overall, our results revealed that the southern region dominated majorly by micro-sized diatoms was positively correlated with PRT, EAA, and EAAI indicating a good protein quality, while the relatively solitary *P. antarctica-*abundant northern region with higher CHO contribution was negatively correlated with good protein quality factors.

## Conclusion

Our study found remarkable differences in biochemical compositions (biomolecular and AA compositions) of the phytoplankton communities (i.e., CHO-rich vs. relatively higher PRT and good vs. bad protein quality) depending upon the growth phase and community structure of phytoplankton. These changes in the biochemical compositions (biomolecular and AA compositions) and protein quality of phytoplankton as a valuable nutrition source could be important for the growth, reproduction, and naupliar survival of herbivorous zooplankton as well as their biochemical composition (Gulati and Demott, 1997; Guisande et al., 2000; Vargas et al., 2006; Yun et al., 2015; Jo et al., 2017). Furthermore, differences in biochemical compositions of POM could also influence the degree of subsequent bacterial degradation and recycling since the lability of individual biochemical compounds varies widely (Harvey et al., 1995; Ingalls et al., 2006; Sabadel et al., 2019; Lehmann et al., 2020). The more refractory compounds could be preserved highly selective with the loss of labile compounds through the microbial process, thereby changing the biochemical compositions of sinking particles and consequently in sediments (Harvey et al., 1995; Alkhatib et al., 2012; Lehmann et al., 2020). Recently, significant changes in physical conditions such as increasing summer temperatures in the atmosphere and surrounding waters were observed in the southwestern Ross Sea continental shelf and lengthening of the free ice season was found in Ross Sea polynya induced by climate change (Stammerjohn et al., 2008; Comiso et al., 2011; Schine et al., 2016; Kaufman et al., 2017). These climate-induced stressors can lead to changes in the size structure and assemblage composition of phytoplankton and physiological shifts in the phytoplankton communities (Yun et al., 2019; Antoni et al., 2020; Hernando et al., 2020). Moreover, herbivores encounter rapidly changing food quality in company with changes in the diverse species, quantity, and biochemical characteristics of their prey (Scott, 1980; Finkel et al., 2010). In addition, differential preservation of biochemical compounds in accordance with reactive changes of the altered biochemical composition of POM under ongoing climate changes could have effects on remineralization rates and sinking particles in the deep sea (Ingalls et al., 2006; Kharbush et al., 2020; Lehmann et al., 2020). Therefore, additional research with multidiscipline approaches is required to evaluate the important food quality as a food source for higher trophic level organisms and understand complicated biochemical parameters associated with climate changes.

## Data Availability Statement

The original contributions presented in the study are included in the article/Supplementary Material. Further inquiries can be directed to the corresponding author/s.

## Author Contributions

SL, NJ, and J-HK contributed to conceptualization. NJ, HL, KK, BK, and MK contributed to data curation. NJ, KK, BK, MK, and WS contributed to sample analysis. NJ, HL, J-HK, BK, and WS contributed to investigation. SL, NJ, and KK contributed to methodology and data validation. HL and J-HK gave scientific advice. NJ and SL contributed to writing—original draft. NJ, HL, and SL contributed to writing—review and editing. All authors agreed with the submission of the manuscript, and read and agreed to the published version of the manuscript.

## Conflict of Interest

The authors declare that the research was conducted in the absence of any commercial or financial relationships that could be construed as a potential conflict of interest.

## Supplementary Material

Supplementary Figure 1 Representative chromatogram for L-amino acids mixed standard; 1: ASP, 2: GLU, 3: ASN, 4: SER, 5: GLN, 6: HIS, 7: GLY, 8: THR, 9: ARG, 10.ALA, 11: TYR, 12: CY2, 13: VAL, 14: MET, 15: NVA, 16: TRP, 17: PHE, 18: ILE, 19: LEU, and 20: LYS.
