# Supplementary figures and images for: Different Biochemical Compositions of Particulate Organic Matter Driven by Major Phytoplankton Communities in the Northwestern Ross Sea
Source: Front Microbiol. 2021 Jan 21;12:623600. doi: 10.3389/fmicb.2021.623600 (PMC7858670; doi:10.3389/fmicb.2021.623600)

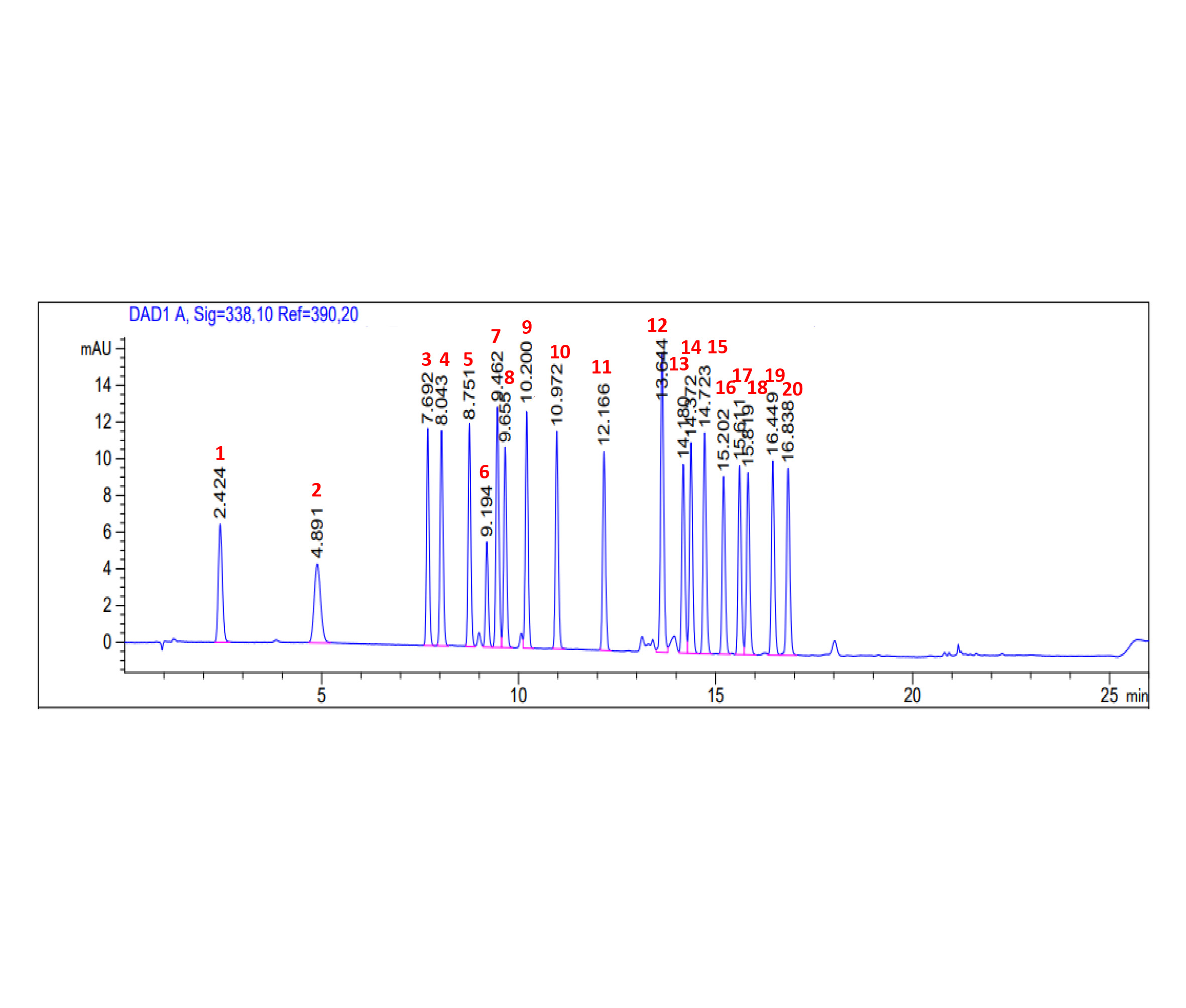

Supplement: Supplementary Figure 1 — Representative chromatogram for L-amino acids mixed standard; 1: ASP, 2: GLU, 3: ASN, 4: SER, 5: GLN, 6: HIS, 7: GLY, 8: THR, 9: ARG, 10.ALA, 11: TYR, 12: CY2, 13: VAL, 14: MET, 15: NVA, 16: TRP, 17: PHE, 18: ILE, 19: LEU, and 20: LYS. [file Image_1.JPEG]
